# Labral Repair Versus Labral Reconstruction in Arthroscopic Treatment of Femoroacetabular Impingement: A Systematic Review and Meta-Analysis

**DOI:** 10.7759/cureus.101348

**Published:** 2026-01-12

**Authors:** Ahmed Elnewishy, Pir Zarak Khan, Yazan Shalan, Saba I Khan, Hagar Teama, Mahmoud Noureldin, Sherin Antony

**Affiliations:** 1 Trauma and Orthopaedics, Royal Berkshire Hospital, Reading, GBR; 2 Trauma and Orthopaedics, Maidstone and Tunbridge Wells NHS Trust, Tunbridge Wells, GBR; 3 Clinical Pharmacy, Faculty of Clinical Pharmacy, Kafrelsheikh University, Kafr El-Sheikh, EGY; 4 Trauma and Orthopaedics, University Hospitals Sussex NHS Foundation Trust, Brighton, GBR

**Keywords:** femoroacetabular impingement, hip arthroscopy, hip outcome scores, hip preservation surgery, irreparable labral tear, labral reconstruction, labral repair, modified harris hip score, revision hip arthroscopy, total hip arthroplasty risk

## Abstract

In the arthroscopic management of femoroacetabular impingement (FAI), labral repair and labral reconstruction are widely used, but their comparative effectiveness remains uncertain. This systematic review and meta-analysis compared functional outcomes, pain scores, revision hip arthroscopy, and conversion to total hip arthroplasty (THA) between labral repair and labral reconstruction in patients undergoing hip arthroscopy for FAI. In November 2025, a systematic search of PubMed, Scopus, Web of Science, Excerpta Medica database (Embase), and the Cochrane Library identified comparative studies reporting extractable postoperative outcomes. Fourteen non-randomized comparative cohorts, including 2,290 hips (1,530 labral repairs and 760 labral reconstructions), met the inclusion criteria. Data were extracted in duplicate, and methodological quality was assessed using the Methodological Index for Non-Randomized Studies (MINORS). A fixed-effect model was used to calculate standardized mean differences (SMDs) and odds ratios (ORs) with 95% confidence intervals (CIs). Labral reconstruction showed a small but statistically significant advantage in postoperative modified Harris Hip Score (mHHS), whereas other functional outcomes, including the Nonarthritic Hip Score (NAHS), Hip Outcome Score Sports Subscale (HOS-SS), and 12-item International Hip Outcome Tool (iHOT 12), were comparable between techniques. Pain improvement measured using the Visual Analog Scale (VAS) was also similar, and revision hip arthroscopy rates did not differ meaningfully between groups. In contrast, labral repair was associated with a significantly lower risk of conversion to THA than labral reconstruction. Heterogeneity across pooled analyses was low, and no relevant publication bias was detected. These findings indicate that both labral repair and labral reconstruction provide clinically important improvement after hip arthroscopy for FAI, with reconstruction performing reliably in irreparable labral pathology, while the lower arthroplasty conversion rate seen with repair likely reflects more favorable baseline joint status in hips where the native labrum can be preserved rather than a direct causal effect of the technique.

## Introduction and background

Femoroacetabular impingement (FAI) is a pathological hip condition caused by abnormal contact between the femoral head-neck junction and the acetabular rim. It leads to hip pain, limited range of motion, and an increased risk of early osteoarthritis. It primarily affects young and middle-aged adults, especially athletes who are exposed to repetitive hip flexion and rotation [1]. FAI is classified into three types: cam (caused by an aspherical femoral head), pincer (caused by acetabular overcoverage), and mixed type, where both cam and pincer mechanisms coexist [2]. The etiology of FAI is multifactorial, involving morphological abnormalities that develop during skeletal maturation and are intensified by high-load adolescent activity, which predisposes individuals to cam deformities [3].

In FAI, an interplay between abnormal bony morphology and joint mechanics leads to elevated contact pressures and shear forces. If left untreated, these abnormal forces can result in labral tears, chondral delamination, and early degenerative changes in the hip joint [4]. Biomechanical analyses have demonstrated altered hip loading patterns, reduced sagittal hip motion, and compensatory pelvic and knee movements during routine activities, consistent with pain-avoidance strategies adopted by patients [5,6]. Importantly, FAI has both mechanical and biochemical dimensions. Recent histological and molecular studies have shown evidence of chronic inflammation and extracellular matrix degradation in impinged hips [7,8].

Since Ganz’s seminal description of FAI in 2003, management has progressively shifted from open surgical hip dislocation to minimally invasive arthroscopic techniques. While open surgical dislocation allows comprehensive deformity correction, it carries greater morbidity, significant soft-tissue disruption, and a potential risk of avascular necrosis of the femoral head [9]. Over the last two decades, arthroscopic techniques have become the preferred approach for symptomatic FAI due to their reduced complication rates, improved intra-articular visualization, and refined instrumentation. Comparative evidence indicates that hip arthroscopy achieves clinical outcomes comparable or superior to open procedures while minimizing morbidity [10]. Furthermore, long-term follow-up studies demonstrate durable symptom relief and joint preservation in over 90% of patients, supporting the widespread adoption of arthroscopy for FAI [11].

Outcomes have been further enhanced by technical refinements in capsular management and portal placement strategies during hip arthroscopy. Capsular suture-lifting techniques and selective capsular plication have been shown to improve joint stability and reduce pain, particularly in patients with borderline dysplasia [12]. Despite variations in surgical technique among surgeons and centers, multi-center data confirm that arthroscopic treatment of FAI yields consistently positive results worldwide [13]. Athletes especially benefit from arthroscopic management, with return-to-sport rates approaching 80%, often reaching pre-injury performance levels within approximately one year after surgery [14]. Moreover, timely arthroscopic intervention may slow the progression toward osteoarthritis and delay the need for total hip arthroplasty (THA) in these patients [15].

Labral preservation is a central principle in arthroscopic FAI management, as the acetabular labrum is essential for hip stability, joint congruency, and maintenance of the suction seal of the joint. Accordingly, when the labral tissue quality is adequate, labral repair has become the preferred strategy. Modern arthroscopic repair techniques utilize suture anchors and advanced suture-passing devices to restore the native labral anatomy, and they consistently outperform simple debridement in terms of functional outcomes and joint preservation [16]. Newer innovations, such as knotless “inversion” repair and controlled-tension anchor systems, facilitate anatomic contour restoration while reducing labral eversion and chondrolabral disruption, thereby improving postoperative recovery [17-19].

Additional surgical techniques have been developed to maintain vascularity and enhance healing at the chondrolabral junction. Approaches such as labral cuff refixation and self-grasping suture passage aim to preserve blood supply and promote healing of the repaired labrum [20,21]. The long-term outcomes after labral repair are very favorable: studies report sustained pain relief, improved hip function, and low rates of conversion to arthroplasty at 7-10 years post-surgery [22]. Notably, even patients with small or hypoplastic labra achieve outcomes with primary repair that are comparable to those with a normal-sized labrum, challenging the need for routine labral reconstruction in such cases [23]. Histological evidence further supports labral repair by demonstrating that repaired labra retain their fibrocartilaginous properties and exhibit revascularized healing tissue [24].

If the labrum is irreparable or severely deficient, however, labral reconstruction becomes necessary. The goal of reconstruction mirrors that of repair: to restore the fluid “suction seal” of the joint, enhance hip stability, and optimize joint biomechanics for the patient [18]. Both autograft and allograft tissues have been successfully used for labral reconstruction, including graft sources such as the iliotibial band, gracilis tendon, and ligamentum teres. In particular, circumferential allograft reconstruction (in which the graft replaces the labrum around the entire acetabular rim) has demonstrated excellent mid-term outcomes and low failure rates in both primary and revision hip arthroscopy settings [25].

Compared to labral debridement, labral reconstruction offers superior clinical results, especially in patients with substantial chondral damage or calcified, nonviable labral tissue. Matched cohort studies have shown that reconstruction is associated with a significantly reduced risk of progression to THA and with improved functional scores when compared to labral debridement alone [26,27]. Additionally, labral reconstruction has been shown to support high return-to-sport rates and durable symptom improvement in athletic populations [18]. Newer techniques, such as complete circumferential labral reconstruction combined with precise acetabular rim recontouring, have reported high procedural success rates and substantial functional gains in patients with severe pincer-type FAI [28]. Capsular autograft reconstruction (using a strip of the patient’s own capsular tissue to reconstruct the labrum) has also shown promising results, offering a cost-effective graft option while maintaining the capsular stability of the hip joint. In current practice, arthroscopic labral repair is generally preferred when the native labrum is structurally viable, whereas labral reconstruction is reserved for irreparable, calcified, or deficient labra in which an effective suction seal cannot be restored by repair alone.

Despite expanding experience with both techniques, the comparative evidence remains uncertain. Published studies are almost entirely non-randomized cohort series, often mixing primary and revision cases, using different indications for reconstruction, and reporting heterogeneous outcome measures. Some cohorts suggest better durability or joint preservation with repair, whereas others report equivalent or even superior outcomes with reconstruction in structurally compromised hips. No randomized controlled trials directly comparing repair and reconstruction have been published. This persistent clinical equipoise and conflicting observational data justify a systematic review and meta-analysis to synthesize the available comparative evidence.

The aim of this review is to compare the clinical and functional outcomes of labral repair versus labral reconstruction in the arthroscopic management of FAI.

## Review

Materials and methods

Search Strategy

This systematic review and meta-analysis was conducted and reported in accordance with the Preferred Reporting Items for Systematic Reviews and Meta-Analyses (PRISMA) 2020 guidelines [29]. A comprehensive literature search was conducted in November 2025 across PubMed, Scopus, Web of Science, Excerpta Medica database (Embase), and the Cochrane Library. The search strategy incorporated both Medical Subject Headings (MeSH) and free-text terms, including “femoroacetabular impingement,” “FAI,” “hip arthroscopy,” “labral repair,” “labral reconstruction,” “labral graft,” and “hip labrum surgery.” To ensure no relevant studies were missed, the reference lists of all eligible full-text articles and related systematic reviews were also screened manually. 

Study Selection: Inclusion and Exclusion Criteria

Studies were selected according to predefined inclusion and exclusion criteria (Table 1). We included prospective or retrospective comparative observational studies that directly compared arthroscopic labral repair with arthroscopic labral reconstruction in patients undergoing hip arthroscopy for FAI. No randomized controlled trials met the eligibility criteria, and all included studies were non-randomized comparative observational cohorts, which have important implications for potential selection bias, residual confounding, and causal interpretation. Eligible studies reported extractable quantitative postoperative data for at least one of the following endpoints: hip-specific functional scores (modified Harris Hip Score (mHHS) [28], Nonarthritic Hip Score (NAHS) [29], Hip Outcome Score Sports Subscale (HOS-SS) [30], 12-item International Hip Outcome Tool (iHOT 12) [31]), pain scores measured with the Visual Analog Scale (VAS), revision hip arthroscopy, or conversion to THA. Only full-text articles published in English were included.

**Table 1:** Inclusion and exclusion criteria for study selection mHHS: modified Harris Hip Score; NAHS: Nonarthritic Hip Score; HOS-SS: Hip Outcome Score Sports Subscale (HOS-SS); iHOT 12: 12-item International Hip Outcome Tool; THA: total hip arthroplasty

Inclusion criteria	Exclusion criteria
Patients undergoing hip arthroscopy for femoroacetabular impingement	No extractable quantitative postoperative outcome data for the prespecified endpoints
Direct comparison of arthroscopic labral repair and arthroscopic labral reconstruction	Mixed hip pathologies without an isolated femoroacetabular impingement subgroup
Reporting at least one eligible postoperative endpoint (mHHS, NAHS, HOS-SS, iHOT 12, VAS, revision arthroscopy, or conversion to THA)	Case series, case reports, cadaveric studies, biomechanical investigations, technical descriptions, narrative reviews, commentaries, or conference abstracts
Full text article available	Overlapping patient cohorts where another publication provided a more complete dataset or longer follow-up
Prospective or retrospective comparative observational design, published in English	

Studies were excluded if they did not directly compare labral repair and labral reconstruction, did not report extractable quantitative postoperative outcomes, included mixed hip pathologies without an isolated FAI subgroup, or were designed as case series, case reports, cadaveric or biomechanical studies, technical notes, reviews, commentaries, or conference abstracts. When multiple publications originated from overlapping patient cohorts, the study with the most complete dataset and longest follow-up was retained.

Outcome Measures

The primary outcomes were postoperative hip-specific functional scores, including the modified Harris Hip Score (mHHS) [28], Nonarthritic Hip Score (NAHS) [29], Hip Outcome Score Sports Subscale (HOS-SS) [30], and 12-item International Hip Outcome Tool (iHOT-12) [31]. Additional outcomes included postoperative pain measured using the VAS, revision hip arthroscopy, and conversion to THA.

Data Extraction and Quality Assessment

Data from each included study were extracted using standardized forms, including details on study design, sample size, patient demographics, surgical techniques (and any concomitant procedures), follow-up duration, and all reported clinical outcome measures. Two independent reviewers performed the data extraction, cross-checked all entries, and resolved any discrepancies through discussion and consensus. Methodological quality and risk of bias for non-randomized comparative studies were assessed using the Methodological Index for Non-Randomized Studies (MINORS), a validated tool for surgical observational research [30]. Each study received a MINORS score and was categorized as low, moderate, or high quality based on previously described thresholds [31].

Statistical Analysis

All meta-analyses were performed using Review Manager (RevMan v5.4, The Cochrane Collaboration, London, UK) [32]. For continuous outcomes, standardized mean differences (SMDs) with 95% CIs were calculated, and for dichotomous outcomes, odds ratios (ORs) with 95% CIs were used. In line with the review protocol and the overall consistency of the datasets, a fixed-effect model was applied for all pooled analyses. Statistical heterogeneity was assessed using the chi-square test and quantified with the I² statistic as described by Higgins and colleagues [33]. Potential publication bias was evaluated through visual inspection of funnel plots and Egger’s regression test for funnel plot asymmetry [34], with a p-value < 0.05 considered statistically significant.

Results

Search Results and Study Selection

The initial database search yielded 312 records across all sources. After removal of 67 duplicate entries, 245 unique records remained for title and abstract screening. Of these, 209 records were excluded during the screening stage for failing to meet the inclusion criteria. Common reasons for exclusion at this stage included the lack of a direct comparison between labral repair and labral reconstruction (e.g., studies reporting only one of the techniques), non-comparative or single-arm study design, mixed patient populations without separate data for FAI, inclusion of non-arthroscopic procedures, or publication types not eligible for inclusion (such as technical notes, cadaveric or biomechanical studies, reviews, and conference abstracts). Additionally, studies that did not report any extractable patient outcome data were excluded during initial screening.

**Figure 1:**
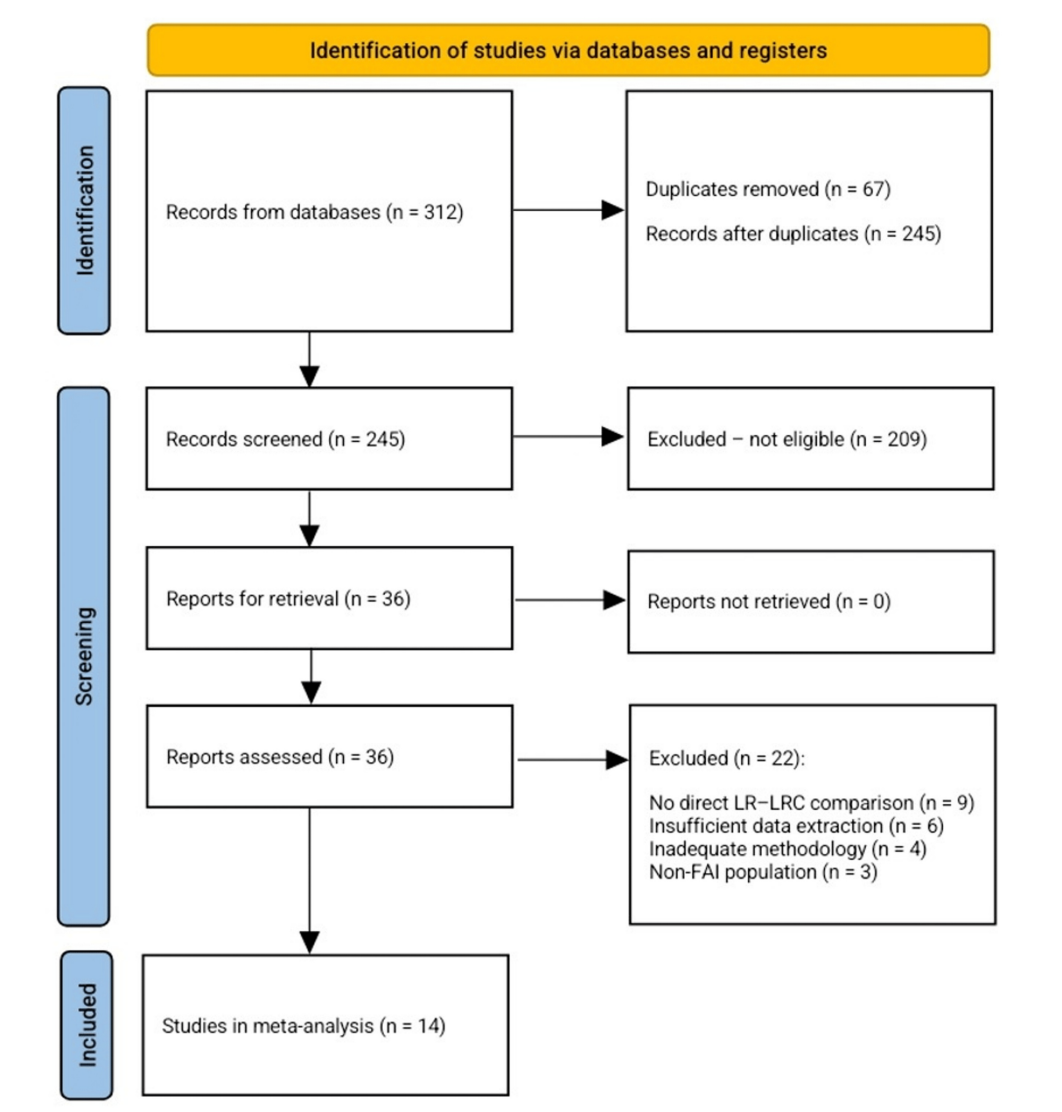
PRISMA flow chart for the included studies PRISMA: Preferred Reporting Items for Systematic Reviews and Meta-Analyses; LR: labral repair; LRC: labral reconstruction; FAI: femoroacetabular impingement

After the initial screening, 36 full-text articles were retrieved and assessed for eligibility. Of these, 22 studies were excluded after full-text review due to reasons such as non-comparative methodology, inadequate length of follow-up, insufficient or non-extractable outcome data, inclusion of patient populations other than FAI, or a lack of clear separation between labral repair and labral reconstruction cohorts in their analysis. Ultimately, 14 studies met all the inclusion criteria and were incorporated into the final qualitative synthesis and quantitative meta-analysis. The complete study selection process is summarized in Figure 1.

Study Characteristics

A total of 2290 hips were analyzed across 14 comparative cohorts, 1530 treated with labral repair and 760 with labral reconstruction. Mostly single-surgeon, single-center, with one multicenter dataset, all studies were Level III. Patients primarily had symptomatic FAI that failed conservative management. Both primary and revision hip arthroscopy populations were included in the cohorts. Revision groups also contained athlete-only and age-specific subgroups. One bilateral design compared repair in one hip versus reconstruction in the other within the same patient.

Reconstruction patients were consistently older and structurally more degenerated across all data. Repair groups generally averaged late 20s to late 30s, while reconstruction groups ranged from late 30s to early 50s, including an example of 52.6 vs 36.5 years. In the mid-20s, kg/m² was the BMI. Although restricted to Tönnis 0-1, reconstruction arms had more Tönnis 1, more severe labral damage, and more grade 3-4 cartilage lesions. Age ≥45 years, BMI ≥23.1 kg/m², and vertical center-edge angle (VCA) ≥36° predictors reduced joint space and required reconstruction. Taken together, patients in the reconstruction cohorts were typically older, had a higher prevalence of Tönnis grade 1 changes, and exhibited more advanced acetabular and femoral cartilage damage at baseline compared with patients treated with labral repair.

Across cohorts, surgical techniques were similar. Repair used suture-anchor refixation of viable labrum, while reconstruction was reserved for irreparable or deficient tissue. Performed as segmental or complete circumferential reconstruction grafts, including gracilis autograft, iliotibial band autograft/allograft, tensor fascia lata allograft, and tibialis anterior allograft. Follow-up durations were adequate: most series had ≥2 years (mean two to three years), with several providing five-year outcomes and registry cohorts reporting median 5.3-5.8 years. Revisions or THA conversions were typically classified as failures. 

Outcome measures were consistent (mHHS, NAHS, HOS-ADL/HOS-SSS, iHOT 12, VAS, 12-Item Short Form Health Survey (SF-12)/Veterans RAND 12-Item Health Survey (VR-12)). Meaningful functional improvement was demonstrated in both groups, with many analyses reporting achievement of the minimal clinically important difference (MCID), patient acceptable symptom state (PASS), maximum outcome improvement (MOI), or substantial clinical benefit (SCB). Complication rates were low. Re-revision ranged from 6% to 20% and THA from 3% to 7% in revision settings. In primary FAI, due to age and more advanced degeneration, THA conversion was generally <5% to 10%, though higher in reconstruction groups. Table 2 summarizes the key characteristics of the included studies.

**Table 2:** Summary of key methodological and clinical characteristics of studies comparing LR and LRC in arthroscopic management of FAI. LR: labral repair; LRC: labral reconstruction; FAI: femoroacetabular impingement; PROs: patient-reported outcomes; mHHS: modified Harris Hip Score; NAHS: Nonarthritic Hip Score; HOS-ADL: Hip Outcome Score – Activities of Daily Living; HOS-SS: Hip Outcome Score – Sports Subscale; iHOT-12: International Hip Outcome Tool-12; VAS: Visual Analog Scale; THA: total hip arthroplasty; MCID: minimal clinically important difference; PASS: patient acceptable symptom state; MOI: maximum outcome improvement; SCB: substantial clinical benefit; FU: follow-up; OA: osteoarthritis; Tönnis: Tönnis Radiographic Grade.

Category	Study Design	Sample Size (Repair/Reconstruction)	Level of Evidence	Patient Demographics	Intervention Details	Follow-up Duration	Outcome Measures	Results	Complications	Conclusion
Bodendorfer et al. [33]	Multicenter retrospective cohort of revision hip arthroscopy (labral repair vs reconstruction)	40 / 55 (total 95)	III	Mean age: 30.0 ± 10.7 (repair) vs 34.4 ± 9.7 (recon); BMI 25.5 ± 5.3 vs 24.1 ± 3.8; ≈70% female	Revision arthroscopy after failed prior surgery for FAI/labral tear; standard FAI correction and chondral work. Repair for viable/reparable labrum; reconstruction (segmental/circumferential graft) for hypoplastic, complex, degenerative, or calcified labrum; rim prepared to bleeding bone and graft anchored to restore seal	Minimum 2 yrs; mean ≈24.8 months	mHHS, HOS-ADL, HOS-SS, iHOT-12, VAS pain & satisfaction; MCID & PASS rates	Both groups improved significantly in all PROs (exact pre-/post means not all reported in abstract). No significant differences between repair and reconstruction in two-year mHHS, HOS-ADL/SS, iHOT-12, VAS pain or satisfaction; MCID/PASS achievement was similar	No hips converted to THA within two years; other specific complications not emphasized	Revision labral repair and labral reconstruction give similar two-year PROs and clinical benefit when chosen according to labral tissue quality/complexity.
Chandrasekaran et al. [34]	Single-surgeon retrospective matched primary FAI cohort (1:2 recon: repair)	68 / 34 (total 102)	III	Mean age: 38.4 ± 12.3 vs 37.3 ± 12.2 yrs; BMI 26.9 ± 4.2 vs 26.9 ± 4.7; similar sex; Tönnis 0–1	Primary hip arthroscopy for FAI; cam/pincer correction, chondral and LT work, capsular management. Repair for reparable labrum; segmental reconstruction (gracilis autograft/allograft) for irreparable, complex, degenerative, ossified, or hypoplastic labrum	Minimum 2 yrs; mean ≈36.8 months (recon) vs 42.8 months (repair)	mHHS, NAHS, HOS-SS, VAS pain, iHOT-12, satisfaction; MCID & PASS (mHHS)	Pre→post (repair vs recon): mHHS 62.9 ± 15.8→85.6 ± 15.4 vs 65.7 ± 17.4→80.4 ± 17.7; NAHS 61.9 ± 19.6→83.8 ± 14.3 vs 61.8 ± 17.6→81.1 ± 16.4; HOS-SS 44.8 ± 27.4→71.9 ± 26.3 vs 43.2 ± 26.0→74.2 ± 24.3; VAS 6.0 ± 2.6→2.4 ± 2.2 vs 5.4 ± 2.6→2.6 ± 2.2. iHOT-12 ≈75.7 vs 68.4 at latest FU; satisfaction similar. PASS mHHS 83.9% vs 70.0%; MCID 79.0% vs 60.0% (NS)	Revision arthroscopy: 11.8% vs 11.8%; THA: 8.8% vs 11.8%; low minor complications	In primary FAI, labral reconstruction for irreparable labra yields similar short-term outcomes and reoperation rates to labral repair for reparable labra.
Domb et al. [35]	Single-surgeon retrospective cohort with matched-pair primary FAI comparison (segmental reconstruction vs repair), ≥5-year follow-up	51/17 in matched primary cohort (overall recon n=23)	III	Age ≈36 yrs; BMI ≈25 kg/m²; sex balanced; FAIS with Tönnis ≤1, LCEA ≥18°, no prior Ipsilateral surgery	Primary hip arthroscopy for FAIS with labral tear; FAI correction, chondral & LT treatment, capsular work. Repair for viable labrum; segmental reconstruction with tendon graft when irreparable/non-viable labrum; graft fixed with anchors on prepared rim	Minimum five years; matched cohort follow-up ≈66–72 months	mHHS, NAHS, HOS-SSS, VAS pain; iHOT-12, SF-12, VR-12, satisfaction; MIC/MCID & PASS for mHHS, HOS-SSS	Overall recon (n=23): mHHS 60.2→80.2 (Δ +17.8), NAHS 55.2→78.8 (Δ +22.0), HOS-SSS 37.3→65.5 (Δ +25.4), VAS 6.0→2.7 (Δ –2.9); PASS mHHS 65%, PASS HOS-SSS 70%. Matched repair vs recon at five years: mHHS 64.6 ± 16.1→87.4 ± 15.3 vs 63.5 ± 18.2→83.9 ± 14.9; NAHS 59.7 ± 19.7→86.9 ± 16.5 vs 57.9 ± 16.1→82.0 ± 16.9; HOS-SSS 40.3 ± 24.8→77.2 ± 23.8 vs 39.5 ± 24.1→69.3 ± 26.0; VAS 6.4 ± 2.2→2.0 ± 2.2 vs 5.9 ± 2.8→2.2 ± 2.2. MCID/PASS similar; satisfaction 8.5 ± 1.7 vs 6.7 ± 3.4	Revision arthroscopy: 7.8% vs 11.8%; THA: 13.7% vs 13.0%	Segmental reconstruction gives durable ≥5-yr improvements comparable to repair, but with slightly lower satisfaction; repair remains preferred when feasible.
Domb et al. [36]	Single-surgeon case–control, propensity-matched primary FAIS comparison of circumferential reconstruction vs repair; ≥2-year follow-up	111 / 37 (total 148)	III	After matching: age 45.6 ± 9.8 vs 45.6 ± 11.6 yrs; BMI 27.2 ± 4.6 vs 27.1 ± 5.0; ≈40–60% male	Primary arthroscopy for FAIS. Repair with base refixation or loop stitch for a viable labrum. Circumferential reconstruction with tibialis anterior allograft (6.5–7.5 mm) for circumferentially calcified/non-viable labrum from 4–6 o’clock; complete labrectomy, rim prep, pull-through graft fixed with knotless anchors; same FAI correction in both	Minimum two years; mean 27.3 ± 3.5 months (repair) vs 25.5 ± 1.6 months (recon)	mHHS, NAHS, HOS-SSS, iHOT-12, VAS pain; SF-12 & VR-12, satisfaction; MCID & PASS (mHHS, iHOT-12)	Recon vs repair pre→post: mHHS 62.9 ± 15.1→86.7 ± 18.4 vs 60.0 ± 15.2→86.3 ± 15.7; NAHS 60.5 ± 16.3→86.2 ± 18.6 vs 60.4 ± 18.2→85.4 ± 16.3; HOS-SSS 38.7 ± 25.1→78.4 ± 27.9 vs 36.8 ± 23.2→74.9 ± 27.0; iHOT-12 34.9 ± 21.7→77.0 ± 28.0 vs 35.0 ± 21.6→77.8 ± 24.3; VAS 5.1 ± 2.1→2.0 ± 2.5 vs 5.1 ± 2.2→2.1 ± 2.5. No significant between-group differences in any PRO or satisfaction (≈8.0–8.1/10); MCID/PASS rates similar (e.g., mHHS PASS ≈80% vs 83%)	Revision arthroscopy: 0% vs 3.6%; THA: 5.4% vs 4.5% (NS); no major complications reported	Circumferential reconstruction for irreparable labra achieves short-term outcomes, MCID/PASS, and failure rates comparable to repair, and is a reliable primary option when the labrum cannot be repaired.
Dornan et al. [37]	Prospective registry, retrospective primary FAI cohort with inverse-propensity weighting (repair vs reconstruction); ≥2-yr, median >5-yr follow-up	At endpoint: 724/129 (PRO analysis 660/108)	III	Reconstruction: more males (67% vs 50%), greater cartilage damage (acetabular grade 3–4: 47% vs 31%; femoral 50% vs 28%), smaller joint space; age and BMI similar (~38–39 yrs; BMI mid-20s)	Single high-volume surgeon. Standard FAI arthroscopy. Repair to restore the suction seal if the labrum is reparable. Reconstruction (mostly segmental graft) when the labrum is deficient, calcified, degenerative, or nonfunctional. Same rehab. Inverse propensity-weighted regression adjusted for age, sex, joint space, alpha angle, LCEA, cartilage & labral morphology, etc.	Minimum 2 years; median 5.3 years (recon) vs 5.8 years (repair); mean ≈6.4–6.6 years	HOS-ADL, HOS-Sport, mHHS, WOMAC, Tegner, SF-12 PCS/MCS, satisfaction; revision arthroscopy & THA	Both groups improved. At median ≈5–6 years: reconstruction vs repair (unadjusted medians) – HOS-ADL ≈90 vs 93, HOS-Sport ≈80 vs 86, WOMAC 10 vs 6 (P<0.05), satisfaction ≈9/10 both. IPTW: reconstruction associated with −3.3 HOS-ADL (95%CI −5.8 to −0.7; P=0.012) and +2.6 worse WOMAC (0.1–5.2; P=0.044); differences in mHHS, SF-12, satisfaction NS	THA: 20% (26/129) recon vs 7% (51/724) repair; OR 3.19 (95%CI 1.17–8.76; P=0.024). Revision arthroscopy 10% vs 6.2% (P=0.108). No major intra-op complication pattern noted	Long-term, repair shows slightly better function (HOS-ADL/WOMAC) and significantly lower THA risk than reconstruction, even though reconstruction is used in more advanced labral/cartilage disease; supports repair as the primary standard when feasible.
Jimenez et al. [38]	Athlete-only cohort of revision hip arthroscopy; prospective registry; 1:1 propensity-matched repair vs reconstruction	30 / 30 in matched subcohort (overall recon athlete n=47)	III	Athletes only. Overall recon: mean age 29.6 ± 9.7 years; 34% male; BMI 25.4 ± 4.5. Matched recon vs repair: age 28.5 ± 10.1 vs 29.9 ± 11.6 years; BMI 25.2 ± 5.0 vs 25.9 ± 4.1; similar sports level	Revision arthroscopy for FAIS after failed index arthroscopy. FAI correction and standard intra-articular work. Reconstruction (mostly allograft, some autograft) for irreparable/calcified/non-viable labrum; revision repair when labrum is still reparable; identical rehab (restricted weightbearing, brace, progressive RTS)	Minimum two years; overall recon mean 31.7 ± 9.3 months; matched recon vs repair 26.3 ± 2.4 vs 30.7 ± 8.6 months	mHHS, NAHS, HOS-SSS, VAS pain, satisfaction; MCID per score; MOIST thresholds; RTS rate; re-revision & THA	In the entire recon cohort (n=41 with PROs): mHHS 58.2 ± 12.3→76.2 ± 18.4 (Δ +17.4), NAHS 56.3 ± 13.5→74.8 ± 18.4 (Δ +16.7), HOS-SSS 31.3 ± 17.3→56.4 ± 25.7 (Δ +24.3), VAS 5.8 ± 2.0→3.5 ± 2.4 (Δ –2.3); satisfaction 6.2 ± 3.3. MCID: mHHS 65%, NAHS 76.9%, HOS-SSS 67.6%, VAS 78%. Matched 30 vs 30 at ≥2 yrs: mHHS 75.7 ± 19.5 vs 77.9 ± 15.9; NAHS 74.9 ± 17.6 vs 79.1 ± 15.8; HOS-SSS 55.1 ± 24.6 vs 59.5 ± 26.9; VAS 3.2 ± 2.4 vs 3.1 ± 1.8; satisfaction 5.9 ± 3.8 vs 7.0 ± 4.1 (all NS); MCID/MOIST rates similar. RTS among those attempting: 63.6% (14/22) recon vs 78.3% (18/23) repair (P=0.337)	Re-revision: 6.7% vs 13.3% (NS); THA 3.3% vs 3.3%; no major other complications reported	In athletes undergoing revision arthroscopy, reconstruction for irreparable labra yields similar PRO gains, MCID/MOIST, RTS, and failure rates as revision repair.
Matsuda et al. [39]	Single-surgeon retrospective primary FAI cohort: labral refixation (repair) vs labral reconstruction (gracilis autograft)	46 / 8 (total 54 hips)	III	Recon: 7/8 male; mean age 34.6 yrs (18–58); BMI 28.4 ± 5.6; 38% Tönnis 1. Repair: mean age 37.5 ± 12.9 yrs (18–73); BMI 27.4 ± 5.3; ≈54% male; 15% Tönnis 1	Arthroscopic FAI correction (acetabuloplasty + femoroplasty) for mixed cam–pincer FAI. Refixation when the labrum is salvageable; reconstruction for a nonsalvageable labrum (≈2 cm defect, severe degeneration/ossification) with ipsilateral gracilis tendon tubularized and fixed by multiple knotless anchors; identical rehab	Minimum 24 months; recon mean ~30 months	Primary: NAHS. Pre-op NAHS 41.9 (recon) vs 55.4 (repair); post-op 92.4 vs 77.9; Δ +50.5 vs +22.5 (P=0.002 for greater improvement with recon). Regression: reconstruction associated with +14.6 higher post-op NAHS (P=0.02); predictive modelling estimated thT recon patients would have ≈15-point lower NAHS if treated with refixation	Two transient pudendal neurapraxias in the recon group; average 2.4 weeks harvest-site knee pain, no permanent deficit. No revisions or THA in the recon group; refixation group had some late adverse events (not strongly quantified)	In carefully selected FAI, labral reconstruction can yield very high NAHS and greater improvement than refixation, with no revisions/THA and acceptable morbidity.	
Nakashima et al. [40]	Single-center retrospective primary FAI case–control: refixation vs ITB autograft segmental reconstruction; plus predictor analysis for unsalvageable labrum	126 / 25 (total 151)	III	Reconstruction older and heavier: age 52.6 ± 15.0 vs 36.5 ± 16.1 yrs; BMI 24.3 ± 2.5 vs 21.8 ± 2.8; more Tönnis 1 (40% vs 17%), higher LCEA & VCA	Primary FAI arthroscopy after >3 months of pain, ROM restriction, positive impingement, and radiographic FAI. Repair if labrum viable & repairable; segmental ITB reconstruction for unsalvageable labrum after debridement; all had acetabular rim trimming, cam osteoplasty, microfracture as needed, and capsular closure	Minimum 2 yrs; mean 37.0 ± 13.2 months (recon) vs 31.9 ± 11.0 months (repair)	mHHS, NAHS; MCID; radiographic predictors (age, BMI, VCA) of unsalvageable labrum; revision & THA	Reconstruction: mHHS 67.3 ± 14.9→95.0 ± 8.1; NAHS 63.0 ± 18.3→89.5 ± 10.1. Repair: mHHS 69.2 ± 18.6→93.0 ± 11.2; NAHS 60.7 ± 18.8→88.6 ± 15.0 (all P<0.001). No significant differences in final mHHS/NAHS between groups; MCID exceeded. Predictors of unsalvageable labrum: age ≥45 yrs (OR 8.83), BMI ≥23.1 (OR 13.05), VCA ≥36° (OR 19.03); ≥2 factors strongly predictive	Revision arthroscopy: 12.0% (3/25) recon vs 11.4% (14/126) repair. THA: 12.0% (3/25) recon vs 0.8% (1/126) repair, mainly older Tönnis 1 with narrowing	Segmental reconstruction gives short-term PROs comparable to refixation but in older, more degenerated hips with higher THA risk; age, BMI, and VCA help anticipate the need for reconstruction.
Perets et al. [41]	Single-surgeon pair-matched revision FAI cohort (1:2 recon:repair)	30 / 15 (total 45)	III	Reconstruction: age 27.0 ± 8.2 yrs; BMI 23.8 ± 3.2; 66.7% prior debridement, 33.3% prior repair. Repair: age 27.5 ± 8.2 yrs; BMI 24.5 ± 3.8; 40% prior debridement, 60% prior repair	Revision arthroscopy for residual pathology after prior hip arthroscopy. Revision repair for unstable but viable labrum; reconstruction (initial gracilis autograft, later semitendinosus allograft) for non-viable, calcified, intrasubstance, diminutive labra; FAI correction, chondral work, and capsular management performed as needed	Minimum 2 yrs; mean 43.2 ± 17.4 months (repair) vs 36.6 ± 16.9 months (recon)	mHHS, NAHS, HOS-SSS, VAS; iHOT-12 & satisfaction; PASS & MCID (mHHS); re-revision & THA	Reconstruction pre→post: mHHS 54.2 ± 16.0→72.0 ± 18.3; NAHS 51.2 ± 17.6→73.9 ± 15.5; HOS-SSS 30.5 ± 22.1→57.3 ± 24.3; VAS 6.2 ± 2.2→3.5 ± 1.9. Repair: mHHS 59.3 ± 16.5→84.1 ± 14.8; NAHS 61.0 ± 16.7→82.5 ± 17.2; HOS-SSS 39.6 ± 25.1→70.5 ± 26.1; VAS 5.8 ± 1.8→2.8 ± 2.2. Final mHHS & iHOT-12 significantly higher in repair; Δ-scores similar. PASS mHHS 60.0% recon vs 76.7% repair; MCID 53.3% vs 76.7% (NS)	Re-revision: 20.0% (3/15) recon vs 6.7% (2/30) repair (NS). THA: 6.7% vs 3.3%. One transient numbness in each group	Revision reconstruction is effective for irreparable labra, but revision repair tends to achieve higher final scores when possible; the magnitude of improvement and failure rates are similar.
Scanaliato et al. [42]	Single-surgeon prospective registry, retrospective primary FAI cohort (repair vs circumferential reconstruction with ITB allograft); IPTW analysis	99 / 63 (total 162)	III	Recon older (43.4 ± 10.7 vs 29.5 ± 11.0 yrs), higher BMI (24.6 vs 23.0), more advanced Tönnis grade (0: 75% vs 91%), more severe labral tearing (severe 68% vs 5%), and higher baseline pain (VAS 49.9 vs 41.5)	Primary arthroscopy for FAI and labral tears after ≥6 months of failed conservative care. Repair for mild/moderate labral tearing, especially in younger cam-dominant patients. Complete circumferential reconstruction using ITB allograft tubularized (6 mm, 85–110 mm), fixed with 9–12 anchors from TAL anteroinferiorly to posteroinferior acetabulum when labrum is irreparable/ossified/deficient or in severe pincer, revision, collagen disorders; capsular closure in all	PRO at ≈24 months; failures (re-op/THA) excluded from PRO analysis	mHHS, iHOT-12, SF-12 Physical, VAS pain & satisfaction	Non-failed hips: Repair mHHS 63.4→88.0 (Δ +24.2), iHOT-12 39.3→71.2 (Δ +31.7), SF-12 37.4→50.0 (Δ +12.7), VAS 41.5→14.1 (Δ –27.7). Recon mHHS 60.2→80.7 (Δ +20.4), iHOT-12 37.8→65.8 (Δ +27.8), SF-12 37.6→47.1 (Δ +9.3), VAS 49.9→23.6 (Δ –25.6). Crude post-op scores favored repair; after IPTW, no significant differences in mHHS, iHOT-12, SF-12, or VAS pain/satisfaction (treatment effect estimates ≈0–4 points, all NS)	Failure (re-op/THA): 5/99 (5%) repair vs 5/63 (8%) recon (P=0.48); no major other complications	Primary circumferential reconstruction can achieve similar short-term PROs and failure rates to repair after adjustment, despite worse baseline disease.
Scanaliato et al. [43]	Single-surgeon primary FAI cohort; repair vs complete circumferential reconstruction; ≥5-yr follow-up with responder analysis	68 / 62 (total 130)	III	Recon older (38.3 ± 11.2 vs 29.9 ± 11.5 yrs), worse labral severity (severe 62.9% vs 5.9%), more cartilage damage (Beck III–IV 12.9% vs 1.5%); BMI slightly lower in recon (23.27 vs 28.35)	Primary FAI arthroscopy; same algorithm as 2018 series. Repair for reparable labra with mild–moderate tearing. Complete circumferential reconstruction with frozen TFL allograft following full labrectomy; multiple anchors around the acetabular rim; standard FAI correction and capsulotomy	Minimum 5 yrs; mean ≈60.1 ± 2.2 months (repair) vs 60.4 ± 1.5 months (recon)	mHHS, iHOT-12, VAS pain & satisfaction; MCID, PASS, MOI, SCB for mHHS & iHOT-12	Repair pre→5 yrs: mHHS 66.10 ± 16.9→83.23 ± 16.3 (Δ +17.13), iHOT-12 39.80 ± 15.8→80.59 ± 13.5 (Δ +40.79), pain VAS 42.33 ± 18.8→24.14 ± 17.4 (Δ –18.19). Recon: mHHS 58.85 ± 17.4→86.28 ± 16.2 (Δ +27.43), iHOT-12 32.84 ± 13.5→79.52 ± 18.3 (Δ +46.68), VAS 47.67 ± 17.1→26.07 ± 16.8 (Δ –21.6). ΔmHHS significantly greater in recon (P=0.04); other differences NS. Responder rates high and similar: e.g., mHHS MCID 95.6% vs 100%, PASS 77.9% vs 79.0%; iHOT-12 MCID 97.1% vs 100%, PASS ≈73.5% vs 72.6%	Revision arthroscopy 2.9% (2/68) vs 4.8% (3/62); THA 1.5% (1/68) vs 1.6% (1/62); no major complications reported	Over ≥5 yrs, complete circumferential reconstruction gives durable PROs, high responder rates, and low failure, comparable to repair despite worse baseline pathology; mHHS improvement is even greater with reconstruction.
White et al. [44]	Single-surgeon retrospective revision cohort: revision repair vs complete reconstruction; failure defined as re-operation	15 / 98 (113 hips; 104 with ≥2-yr FU)	III	Mean age 34 yrs; revision repair younger (27.8 ± 11.8 yrs) vs recon 34.6 ± 10.2 yrs; similar sex; recon with worse acetabular cartilage (more grade 2–3)	Revision arthroscopy for labral re-tear and residual FAI after prior arthroscopic labral debridement/repair. Early practice: revision repair; later: complete ITB allograft reconstruction. Revision repair: re-repair of the labrum with circumferential sutures. Revision reconstruction: full labrectomy, acetabular rim prep, circumferential ITB allograft fixed front-to-back; FAI correction in both	Minimum 2 yrs; mean 2.6 yrs overall; repair followed longer (4.7 yrs) vs recon (2.4 yrs)	MHHS, LEFS, VAS pain, satisfaction in non-failed hips; primary endpoint failure = further ipsilateral arthroscopic or THA	Failure: 7/14 (50%) revision repair vs 11/90 (12%) reconstruction; crude RR ≈4.1; adjusted RR ≈2.6 (NS but trend). Among non-failed hips, MHHS 56.1 ± 9.1→84.1 ± 18.9 (repair) vs 49.3 ± 16.7→81.2 ± 20.7 (recon); LEFS 49.7 ± 16.0→69.6 ± 13.8 vs 36.8 ± 16.4→62.6 ± 17.0; VAS pain decrease ~2.8 vs 3.6; satisfaction 7.6 vs 8.4; differences NS	18/113 hips (17%) had further surgery (10 further reconstructions, six THA, one debridement, one unknown); no major peri-op complications (infection, fracture, instability) were highlighted	In revision settings, complete reconstruction has a much lower failure rate than revision repair, with similar PROs in surviving hips; reconstruction is favored for failed labral treatment.
White et al. [45]	Self-controlled bilateral primary FAI cohort: each patient had repair in one hip, reconstruction in the other	29 / 29 (58 hips, 29 patients)	III	23 females, 6 males; mean age 32.6 yrs. Reconstruction hips had worse labral and acetabular cartilage at baseline, but similar radiographic alignment and joint space	Bilateral symptomatic FAI without prior surgery. One hip was treated earlier with primary labral repair, and later the contralateral hip with complete reconstruction using ITB allograft. FAI correction in both; same surgeon & rehab	Mean 56 months (repair) vs 40 months (recon)	MHHS, LEFS, VAS pain, satisfaction; patient preference; failure = further hip surgery	Failure: 0/29 (0%) reconstruction vs 9/29 (31%) repair (P<0.01). In non-failed hips: both sides had large improvements; MHHS ≈54→~87; LEFS ≈42–46→~68–69; VAS ≈6→~2.4–2.8; satisfaction ≈8.6–8.7/10; no significant differences in PROs between repair and reconstruction	All reoperations occurred in repaired hips (revised mainly to reconstruction); no major perioperative complications were reported	In bilateral patients, primary reconstruction had 0% failure and similar PROs to repair, suggesting more predictable durability, especially when tissue quality is poorer.
White et al. [46]	Single-surgeon retrospective primary FAI cohort: repair vs reconstruction in ≥40 yrs, and reconstruction ≥40 vs 30–39 yrs	93 / 158 (≥40 yrs comparison); plus 112 recon (30–39 yrs)	III	Reconstruction ≥40 yrs: age 48.1 ± 5.4 yrs; repair ≥40 yrs: 47.0 ± 4.7 yrs; reconstruction 30–39 yrs: 34.6 ± 2.9 yrs. Baseline mHHS: 50.8 (recon ≥40), 51.5 (recon 30–39), 57.8 (repair ≥40); LEFS: 36.4, 39.3, 45.0; VAS ≈6.3, 6.4, 5.7	Primary hip arthroscopy for FAIS/labral pathology after failed non-operative care; preserved joint space, minimal cartilage damage, CE angle ≥25°. Early practice: repair; later: routine complete ITB allograft reconstruction. Repair preserving native labrum; complete reconstruction front-to-back with slightly over-length ITB graft; FAI correction in all	Mean FU ≈4.2 yrs (3.8 recon ≥40; 5.6 repair ≥40; 3.6 recon 30–39); minimum 2 yrs	mHHS, LEFS, VAS pain, satisfaction; MCID mHHS; failure = revision/THA	In non-failed hips: reconstruction ≥40 yrs mHHS 51.1→87.7 (Δ +36.6), LEFS 36.0→68.9 (Δ +33.1), VAS 6.3→2.2 (Δ –4.2); repair ≥40 yrs 60.0→88.3 (Δ +28.3), LEFS 48.5→70.9 (Δ +22.5), VAS 5.5→2.0 (Δ –3.5); reconstruction 30–39 yrs 51.5→89.7 (Δ +38.3), LEFS 40.2→72.1 (Δ +31.8), VAS 6.4→2.2 (Δ –4.0). Post-op mHHS/LEFS/VAS & satisfaction similar across groups (~mHHS high-80s, VAS ≈2, satisfaction ≈8.4–8.6). MCID for mHHS: 76% (recon ≥40), 83% (recon 30–39), 71% (repair ≥40)	Overall failures 11.2% (35/312). Failure rates: 7.4% (10/136) recon ≥40, 8.5% (8/94) recon 30–39, 20.7% (17/82) repair ≥40. Adjusted RR of failure for repair ≥40 vs recon ≥40: 3.29 (95%CI 1.25–8.69; P=0.02). THA ≈3.7–5.3% in all groups	In ≥40 yrs with preserved joint space, complete reconstruction yields greater average mHHS improvement, lower failure rates, and similar final PROs compared with repair. Reconstruction results in ≥40 yrs are comparable to those in 30–39 yrs, supporting reconstruction as a strong option in older, structurally preserved hips.

Quality Assessment of Included Studies

Using MINORS, the methodological quality of the included non-randomized comparative studies was evaluated. The comparative MINORS framework comprises 12 domains, including clarity of study aim, inclusion of consecutive patients, prospective data collection, appropriateness of clinical endpoints, adequacy of follow-up, minimization of loss to follow-up, baseline comparability between groups, and suitability of statistical analyses. Each domain was scored as 0 (not reported), 1 (reported but inadequate), or 2 (reported and adequate), generating a maximum score of 24 per study.

Overall methodological quality was moderate across the 14 included studies, with total MINORS scores ranging from 16 to 20. Most investigations demonstrated clearly defined aims, clinically appropriate endpoints, and adequate follow-up durations. More recent cohorts employing propensity matching or inverse-probability weighting displayed stronger methodological rigor, particularly in group comparability and statistical adjustment. The absence of prospective sample-size calculations, incomplete reporting of consecutive patient enrollment, and limited blinding of outcome assessment were included as consistent limitations. Nevertheless, no study exhibited a pattern of deficiencies consistent with a high overall risk of bias. The detailed MINORS quality assessment for all included studies is presented in Table 3.

**Table 3:** Quality assessment of the included studies using the MINORS tool MINORS: Methodological Index for Non-randomized Studies

Study	Clearly Stated Aim	Inclusion of Consecutive Patients	Prospective Data Collection	Endpoints Appropriate to Aim	Unbiased Assessment of Endpoints	Adequate Follow-up Period	Loss to Follow-up <5%	Prospective Sample-size Calculation	Adequate Control Group	Contemporary Groups	Baseline Equivalence	Adequate Statistical Analyses	Total (0–24)
Bodendorfer et al. [33]	2	1	1	2	1	2	1	0	2	2	1	1	16
Chandrasekaran et al. [34]	2	2	1	2	1	2	1	0	2	2	2	1	18
Domb et al. [35]	2	2	2	2	1	2	1	0	2	1	2	1	18
Domb et al. [36]	2	2	2	2	1	2	1	0	2	2	2	1	19
Dornan et al. [37]	2	2	2	2	1	2	1	0	2	1	2	2	19
Jimenez et al. [38]	2	1	2	2	1	2	1	0	2	1	2	2	18
Matsuda et al. [39]	2	1	1	2	1	2	1	0	2	1	1	2	16
Nakashima et al. [40]	2	2	1	2	1	2	1	0	2	2	1	2	18
Perets et al. [41]	2	1	1	2	1	2	1	0	2	1	2	1	16
Scanaliato et al. [42]	2	2	2	2	1	2	1	0	2	2	2	2	20
Scanaliato et al. [43]	2	2	2	2	1	2	1	0	2	2	1	2	19
White et al. [44]	2	2	2	2	1	2	1	0	2	0	1	1	16
White et al. [45]	2	0	2	2	1	2	2	0	2	0	2	1	16
White et al. [46]	2	2	2	2	1	2	1	0	2	1	1	2	18

Results of the Meta-Analysis

**Figure 2:**
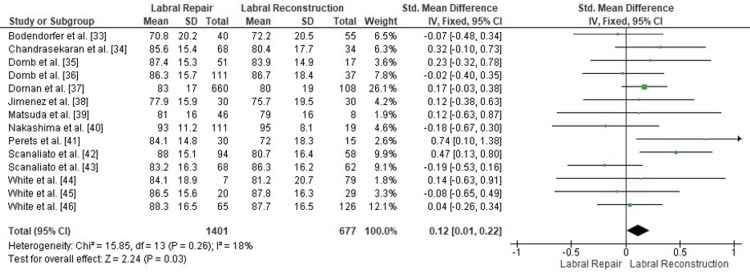
Forest plot comparing postoperative mHHS between labral repair and labral reconstruction. SMD: standardized mean difference; CI: confidence interval; mHHS: modified Harris Hip Score

Comparison of postoperative mHHS between labral repair and labral reconstruction: A pooled analysis of postoperative mHHS demonstrated a small but statistically significant advantage for labral reconstruction over labral repair (SMD = 0.12, 95% CI: 0.01-0.22; p = 0.03). Heterogeneity was low (I² = 18%, p = 0.26), indicating consistency across the included studies and supporting the stability of the effect estimate (Figure 2). These findings suggest that labral reconstruction may provide a modest improvement in functional recovery compared with repair across the available evidence.

**Figure 3:**
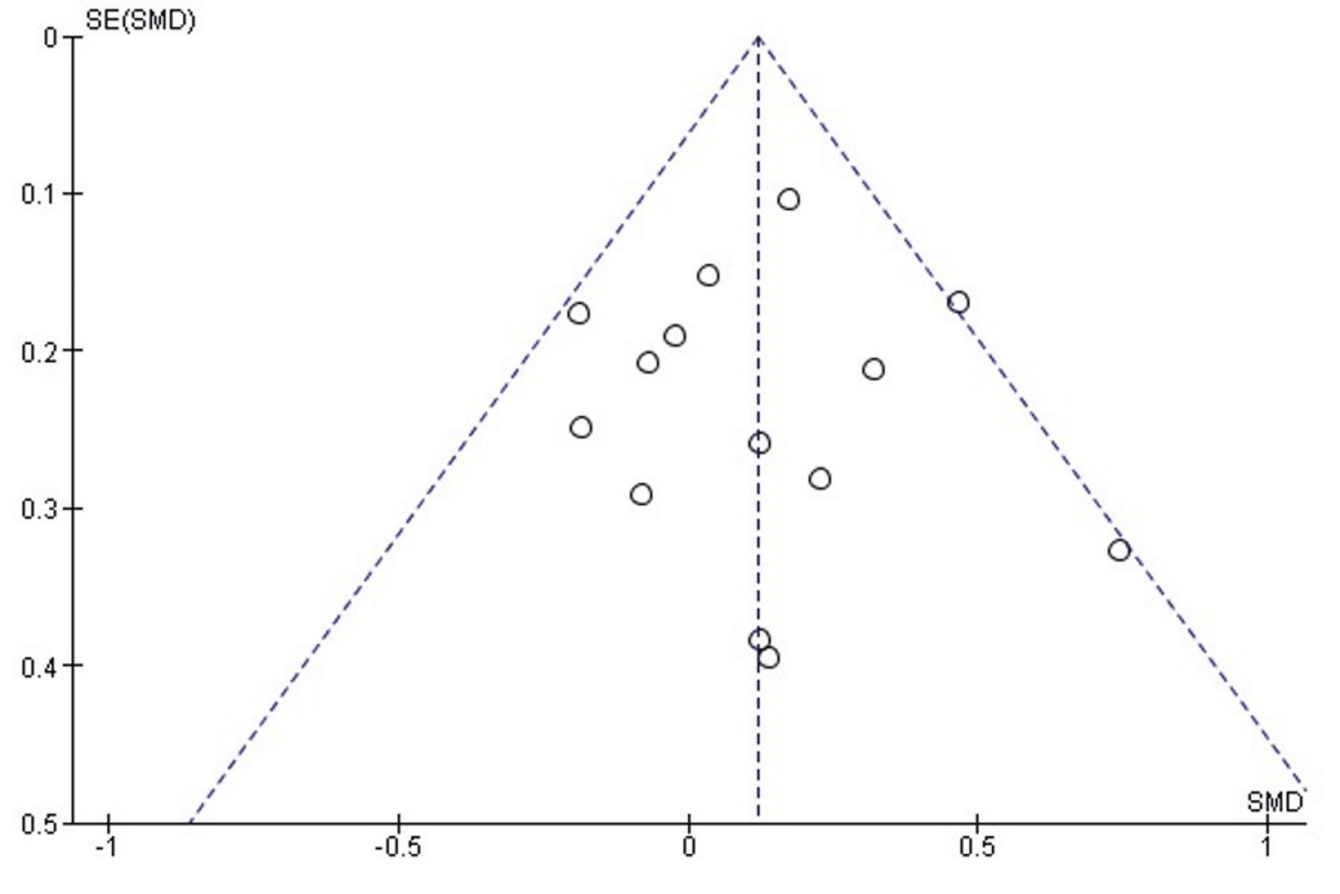
Funnel plot assessing publication bias for postoperative mHHS comparison. SE: standard error; SMD: standardized mean difference; mHHS: modified Harris Hip Score

Publication bias assessment: The funnel plot (Figure 3) demonstrated a generally symmetrical distribution of study estimates, suggesting minimal small-study effects. Egger’s regression test was not statistically significant (p > 0.05), indicating that publication bias is unlikely to have influenced the pooled results.

**Figure 4:**
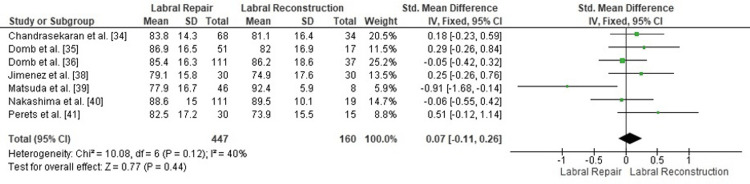
Forest plot comparing postoperative NAHS between labral repair and labral reconstruction. SMD: standardized mean difference; CI: confidence interval; NAHS: Nonarthritic Hip Score

Comparison of postoperative NAHS between labral repair and labral reconstruction: A pooled analysis of postoperative NAHS demonstrated no statistically significant difference between labral repair and labral reconstruction (SMD = 0.07, 95% CI: -0.11 to 0.26; p = 0.44). Heterogeneity was moderate (I² = 40%, p = 0.12), suggesting some variability among studies but not sufficient to compromise the interpretation of the pooled estimate (Figure 4). Overall, these findings indicate that both techniques yield comparable postoperative functional outcomes when assessed using the NAHS metric.

**Figure 5:**
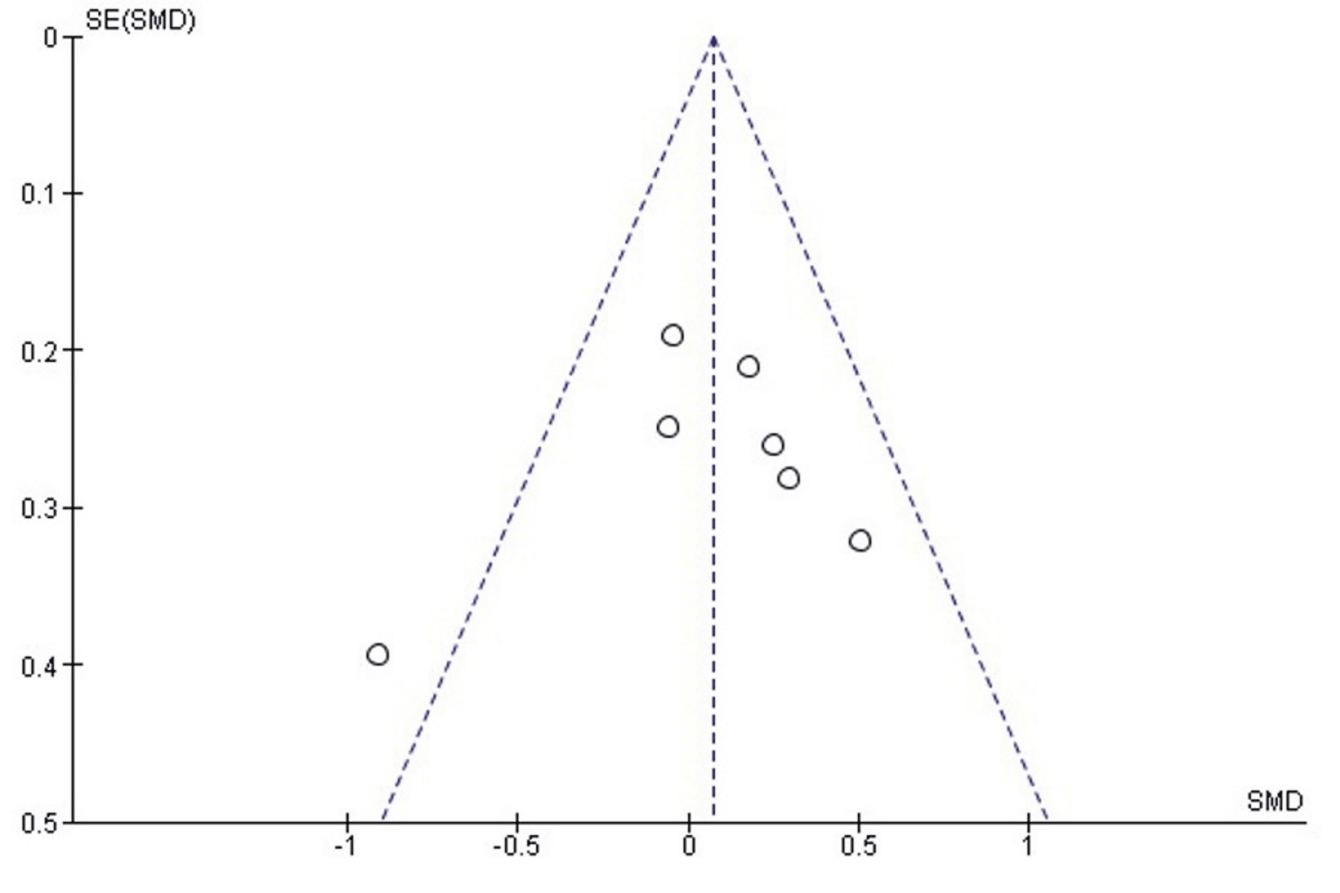
Funnel plot assessing publication bias for postoperative NAHS comparison. SE: standard error; SMD: standardized mean difference; NAHS: Nonarthritic Hip Score

Publication bias assessment: The funnel plot (Figure 5) displayed a generally symmetrical distribution of study estimates, with no pattern suggesting small-study effects. Egger’s regression test was not statistically significant (p > 0.05), indicating that publication bias is unlikely to influence the pooled effect for this outcome.

**Figure 6:**
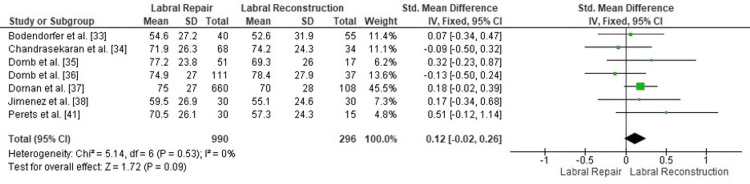
Forest plot comparing postoperative HOS-SS between labral repair and labral reconstruction. SMD: standardized mean difference; CI: confidence interval; HOS-SS: Hip Outcome Score-Sports Subscale

Comparison of postoperative HOS-SS between labral repair and labral reconstruction: A pooled analysis of postoperative HOS-SSS demonstrated no statistically significant difference between labral repair and labral reconstruction (SMD = 0.12, 95% CI: -0.02 to 0.26; p = 0.09). Heterogeneity was negligible (I² = 0%, p = 0.53), indicating excellent consistency among the included studies and supporting the robustness of the pooled estimate (Figure 6). These findings suggest that both procedures yield comparable sports-related functional outcomes following arthroscopic management of FAI.

**Figure 7:**
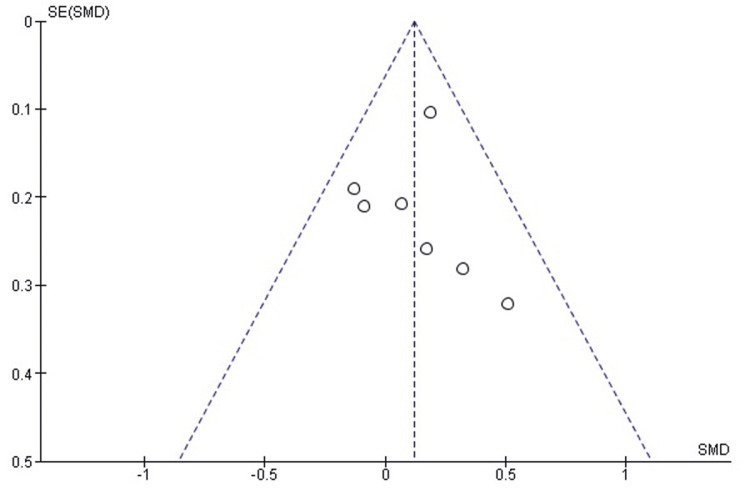
Funnel plot assessing publication bias for postoperative HOS-SS comparison. SE: standard error; SMD: standardized mean difference; HOS-SS: Hip Outcome Score-Sports Subscale

Publication bias assessment: The funnel plot (Figure 7) showed a symmetrical distribution of study estimates, with no indication of small-study effects. Egger’s regression test was not statistically significant (p > 0.05), suggesting that publication bias is unlikely to have influenced the pooled results for HOS-SS.

**Figure 8:**
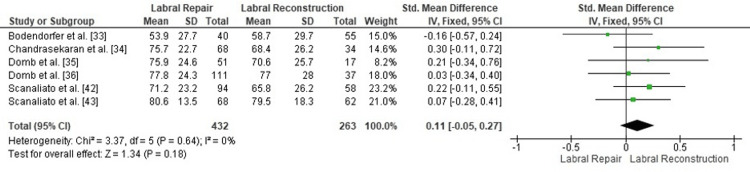
Forest plot comparing postoperative iHOT-12 between labral repair and labral reconstruction. SMD: standardized mean difference; CI: confidence interval; iHOT-12: International Hip Outcome Tool-12

Comparison of postoperative iHOT-12 between labral repair and labral reconstruction: A pooled analysis of postoperative iHOT-12 scores demonstrated no statistically significant difference between labral repair and labral reconstruction (SMD = 0.11, 95% CI: -0.05 to 0.27; p = 0.18). Heterogeneity was negligible (I² = 0%, p = 0.64), indicating excellent consistency among the included studies and supporting the stability of the pooled estimate (Figure 8). These findings suggest that both procedures yield comparable patient-reported functional outcomes when evaluated using the iHOT-12.

**Figure 9:**
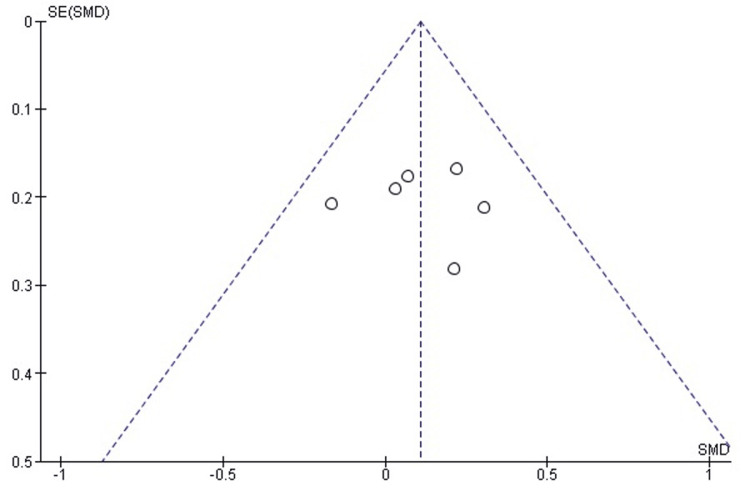
Funnel plot assessing publication bias for postoperative iHOT-12 comparison. SE: standard error; SMD: standardized mean difference; iHOT-12: International Hip Outcome Tool-12

Publication bias assessment: The funnel plot (Figure 9) demonstrated a symmetrical distribution of study estimates, with no indication of small-study effects. Egger’s regression test was not statistically significant (p > 0.05), suggesting that publication bias is unlikely to have influenced the results for the iHOT-12 outcome.

**Figure 10:**
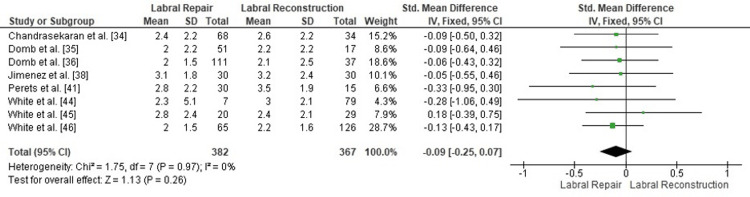
Forest plot comparing postoperative VAS pain between labral repair and labral reconstruction. SMD: standardized mean difference; CI: confidence interval; VAS: Visual Analog Scale

Comparison of postoperative VAS pain between labral repair and labral reconstruction: A pooled analysis of postoperative VAS pain scores demonstrated no statistically significant difference between labral repair and labral reconstruction (SMD = -0.09, 95% CI: -0.25 to 0.07; p = 0.26). Heterogeneity was negligible (I² = 0%, p = 0.97), indicating excellent consistency among the included studies and supporting the stability of the pooled estimate (Figure 10). These findings suggest that both techniques achieve comparable postoperative pain reduction following arthroscopic management of FAI.

**Figure 11:**
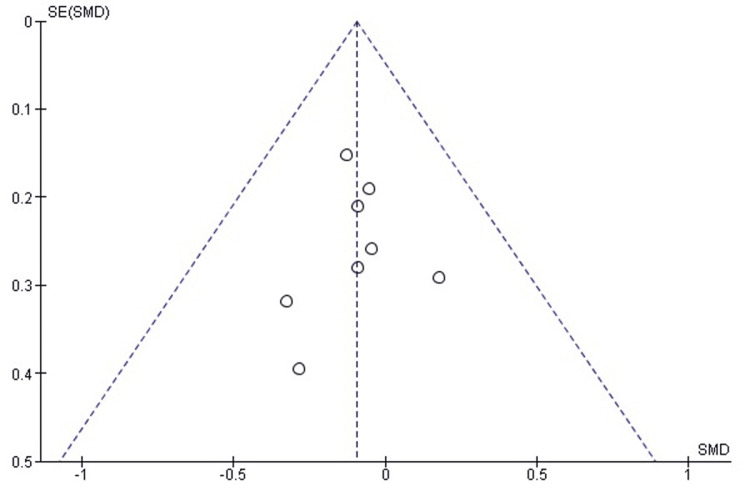
Funnel plot assessing publication bias for postoperative VAS pain comparison. SE: standard error; SMD: standardized mean difference; VAS: Visual Analog Scale

Publication bias assessment: The funnel plot (Figure 11) revealed a symmetrical distribution of study estimates, with no visible pattern indicating small-study effects. Egger’s regression test was not statistically significant (p > 0.05), suggesting that publication bias is unlikely to have influenced the pooled results for VAS pain.

**Figure 12:**
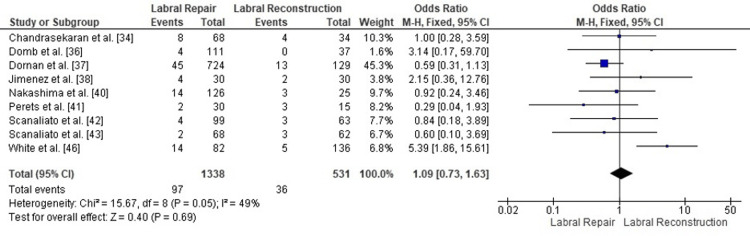
Forest plot comparing revision hip arthroscopy rates between labral repair and labral reconstruction. OR: odds ratio; CI: confidence interval

Comparison of revision hip arthroscopy between labral repair and labral reconstruction: A pooled analysis of revision hip arthroscopy rates demonstrated no significant difference between labral repair and labral reconstruction (OR = 1.09, 95% CI: 0.73 to 1.63; p = 0.69). Heterogeneity was moderate (I² = 49%, p = 0.05), indicating some variability among studies but not enough to undermine the pooled effect estimate (Figure 12). Overall, the findings suggest that both procedures are associated with comparable rates of subsequent revision surgery following arthroscopic treatment of FAI. However, reconstruction cohorts consistently included older patients with more advanced chondral and labral degeneration at baseline, so the lower THA conversion rate in repair groups is likely driven in part by selection bias and underlying joint status rather than a purely technical advantage of repair.

**Figure 13:**
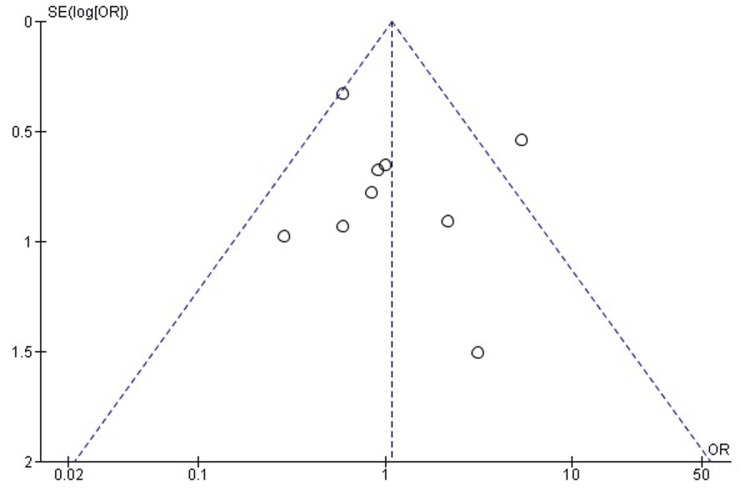
Funnel plot assessing publication bias for revision hip arthroscopy comparison. SE: standard error; OR: odds ratio

Publication bias assessment: The funnel plot (Figure 13) demonstrated a largely symmetrical distribution of study estimates, with no directional clustering or pattern suggesting small-study effects. Egger’s regression test was not statistically significant (p > 0.05), indicating that publication bias is unlikely to have influenced the pooled results for revision hip arthroscopy.

**Figure 14:**
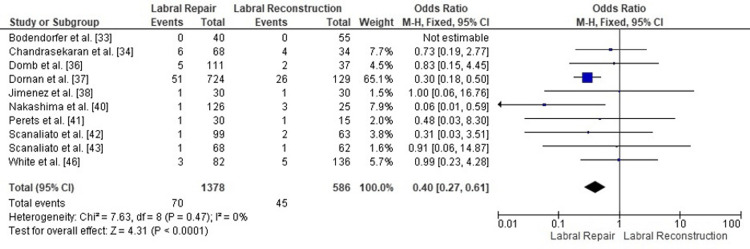
Forest plot comparing conversion to THA between labral repair and labral reconstruction. OR: odds ratio; CI: confidence interval; THA: total hip arthroplasty

Comparison of conversion to THA between labral repair and labral reconstruction: A pooled analysis of conversion to THA demonstrated a significantly lower risk for patients treated with labral repair compared with those undergoing labral reconstruction (OR = 0.40, 95% CI: 0.27 to 0.61; p < 0.0001). Heterogeneity was negligible (I² = 0%, p = 0.47), indicating excellent consistency across the included studies and supporting the robustness of the pooled estimate. These findings suggest that labral repair may be associated with a substantially reduced likelihood of progressing to THA following arthroscopic treatment of FAI (Figure 14).

**Figure 15:**
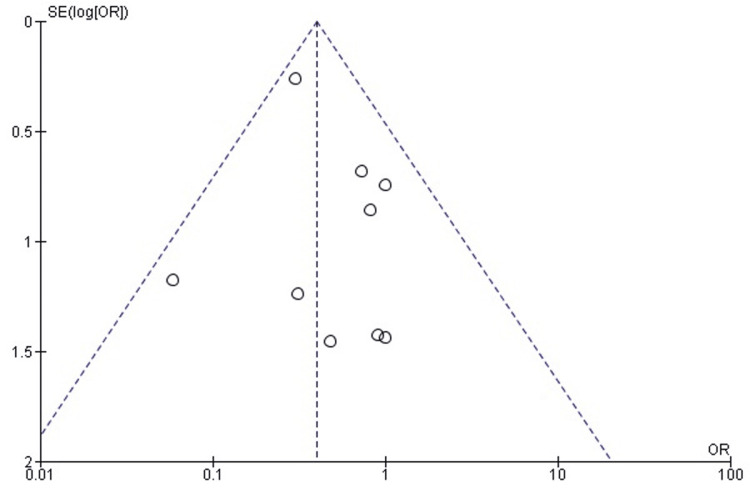
Funnel plot assessing publication bias for conversion to THA comparison. SE: standard error; OR: odds ratio; THA: total hip arthroplasty

Publication bias assessment: The funnel plot (Figure 15) showed a generally symmetrical distribution of study estimates, with no visual evidence of small-study effects. Egger’s regression test was not statistically significant (p > 0.05), indicating that publication bias is unlikely to have influenced the pooled findings for THA conversion.

Discussion

This systematic review and meta-analysis shows that both arthroscopic labral repair and labral reconstruction provide substantial clinical benefit in the management of FAI, with broadly comparable improvements in hip function, pain, and sports-related activity. Across the 14 comparative cohorts, patient-reported outcomes improved well beyond minimal clinically important difference thresholds in most patients, regardless of whether the labrum was repaired or reconstructed [33-43]. Reconstruction was frequently reserved for older patients, revision cases, or hips with irreparable or calcified labra, yet still produced results that approached those of repair in less compromised joints [33-43]. Taken together, these findings support a treatment strategy driven primarily by labral tissue quality, chondral status, and long-term joint preservation goals rather than by an expectation that one technique will consistently outperform the other on standard outcome scores.

The pooled analysis demonstrated a small but statistically significant advantage in postoperative mHHS for labral reconstruction. This effect was largely driven by series in which reconstruction was used for nonfunctional or degenerative labra, where large absolute gains in function were observed despite worse baseline pathology [39,40,43,46]. Although this suggests that reconstruction can deliver meaningful functional recovery even in structurally challenging hips, the magnitude of the pooled difference was modest and likely below the minimal clinically important difference for the mHHS. Furthermore, other comparative cohorts did not confirm a clear superiority of reconstruction. In particular, some studies reported slightly higher final mHHS values after repair, even though reconstruction was used in more diseased joints [37,41]. Across the wider body of evidence, many matched or adjusted analyses found no clinically relevant difference between techniques in medium-term functional outcomes [33-36,38,42,44,45].

Evidence from other functional instruments reinforces the overall impression of equivalence. Scores such as the NAHS, HOS-SS, and iHOT-12 improved substantially after both labral repair and reconstruction in multiple cohorts, including athletic and high-demand populations [34-36,38,40-43]. Where isolated studies suggested directional differences, these were often explained by differences in baseline anatomy, revision status, or labral viability rather than the reconstructive strategy itself [39,41]. When combined with adequate correction of cam and pincer morphology and appropriate capsular management, both approaches appear capable of restoring hip-specific function and quality of life to a similar level for the majority of patients.

Postoperative pain outcomes were likewise similar between techniques. Most comparative series reported large and clinically meaningful reductions in VAS pain scores after both labral repair and reconstruction, without a consistent difference between groups [34-36,38,41-46]. These observations support the concept that pain relief after arthroscopic treatment of FAI is driven mainly by comprehensive correction of the impingement morphology, appropriate management of chondral and subchondral pathology, and reestablishment of a stable labral suction seal. Whether that seal is restored by refixation of a viable native labrum or by a graft appears less important than the overall quality of bony correction and soft tissue balancing.

Reoperation patterns provide additional insight into the durability of each strategy. Rates of revision hip arthroscopy were broadly comparable between labral repair and reconstruction in most comparative cohorts, even when reconstruction was used in more complex or revision settings [34-36,38,40-43]. However, some series suggest that reconstruction may be advantageous in specific high-risk scenarios. In revision surgery, complete labral reconstruction has been associated with lower failure rates than repeat repair, particularly when the labrum is structurally deficient or has failed previous treatment [44]. In bilateral and older patient cohorts, reconstruction has also been reported to have fewer subsequent failures than repair when used in joints with preserved joint space but compromised labral tissue [45,46].

In contrast, the present meta-analysis found that labral repair was associated with a significantly lower risk of conversion to THA compared with reconstruction. This pattern is consistent with larger registry and cohort data in which reconstruction groups were typically older, had more advanced cartilage damage, and demonstrated higher rates of arthroplasty over time [37,40,42,43,46]. It is likely that this difference in THA conversion reflects, at least in part, underlying disease severity and patient selection rather than the labral technique alone. Even so, the pooled estimate suggests that where a healthy, reparable labrum is present, repair may offer the most joint-preserving option in the long term. Reconstruction, by contrast, appears to provide a reliable means of restoring function and delaying arthroplasty in patients who historically might have been managed with debridement or early replacement surgery because the labrum was unsalvageable. These data suggest that repair may remain the more joint-preserving option when a healthy, reparable labrum is present, while reconstruction reliably restores function in more complex cases, but this association should not be interpreted as a causal effect of labral repair, given the nonrandomized designs and baseline differences between cohorts.

From a practical perspective, these findings support an algorithmic, tissue-quality-based approach. Primary labral repair should remain the standard of care when the labrum is structurally viable and can be restored to the anatomic position with a stable suction seal. Labral reconstruction should be considered when the labrum is hypoplastic, calcified, degenerative, or otherwise irreparable, and in revision cases where previous debridement or repair has failed [47-51]. Predictors such as age, BMI, and radiographic measures of acetabular coverage can help anticipate the likelihood of an unsalvageable labrum and guide preoperative counselling [52-54]. Within this framework, reconstruction complements rather than replaces repair, extending the indications for hip preservation surgery to more advanced or complex presentations while maintaining outcomes that are comparable to those of repair in less diseased joints.

Limitations

This review has several limitations that should be considered when interpreting the findings. All included studies were nonrandomized comparative cohorts, which introduces the risk of selection bias and confounding by indication, particularly because reconstruction was preferentially used in older patients and in hips with more severe labral and chondral damage. Surgical techniques also varied between studies with respect to graft choice, fixation methods, capsular management, and rehabilitation protocols. Outcome reporting was not fully uniform, and not all cohorts provided complete data for every functional or radiological endpoint, which limited some subgroup analyses. Finally, although several studies reported medium-term or longer follow-up, data beyond 10 years remain sparse, and the true long-term impact of labral repair versus reconstruction on joint preservation and arthroplasty risk will require further prospective, ideally randomized or carefully matched studies. In addition, despite low statistical heterogeneity for many pooled outcomes, there was meaningful clinical heterogeneity across cohorts in terms of indication for reconstruction, primary versus revision surgery, graft selection, and patient demographics.

## Conclusions

In the setting of arthroscopic treatment for FAI, the present meta-analysis highlights that both labral repair and labral reconstruction achieve substantial and largely comparable clinical improvement in hip function, pain relief, and sports-related activity. Even in more complex cases with irreparable or degenerative labra, reconstruction provides outcomes similar to repair, supporting its role as a reliable joint-preserving option when native tissue quality is inadequate. For reconstruction on mHHS, the small functional advantage observed appears modest and was not consistently mirrored across other scores, whereas labral repair was associated with a lower risk of conversion to THA, suggesting superior long-term joint preservation when the labrum is reparable. These findings indicate that both techniques are effective, and the choice should be individualized according to labral tissue quality, patient characteristics, and intraoperative findings between repair and reconstruction rather than an expectation of major differences in short- to mid-term clinical outcomes.
